# Tumor-derived IL-8 facilitates lymph node metastasis of gastric cancer via PD-1 up-regulation in CD8^+^ T cells

**DOI:** 10.1007/s00262-022-03223-3

**Published:** 2022-05-28

**Authors:** Xiang Li, Jing Zhai, Yuke Shen, Tiancheng Zhang, Yaohui Wang, Yani He, Qiang You, Lizong Shen

**Affiliations:** 1 Department of Surgical Oncology, Jiangsu Province Hospital of Chinese Medicine, Affiliated Hospital of Nanjing University of Chinese Medicine, Nanjing, 210029 China; 2 Digestive Endoscopy Center, Jiangsu Province Hospital of Chinese Medicine, Affiliated Hospital of Nanjing University of Chinese Medicine, Nanjing, 210029 China; 3 Department of Pathology, Jiangsu Province Hospital of Chinese Medicine, Affiliated Hospital of Nanjing University of Chinese Medicine, Nanjing, 210029 China; 4 Department of Geriatrics, Second Affiliated Hospital, Nanjing Medical University, Nanjing, 210003 China

**Keywords:** Gastric cancer, IL-8, Lymph node metastasis, PD-1, T cell exhaustion

## Abstract

**Background:**

The pretherapeutic serum interleukin-8 (sIL-8) levels have been revealed to be increased in about half of patients with locally advanced gastric cancer. However, the roles of IL-8 in lymph node metastasis (LNM) and the underlying mechanisms remain unclear.

**Methods:**

146 patients with primary gastric carcinoma were enrolled in this study. ELISA was used to measure IL-8 levels. The CD4/CD8 ratio and programmed cell death-1 (PD-1) expression of T cells in primary tumor tissues, tumor-draining lymph nodes (TDLNs) and non-draining lymph nodes (NDLNs) were assayed with flow cytometry. Protein expression of the molecules was determined with immunohistochemistry, western blotting or immunoprecipitation. The gastric cancer mouse tumor model with LNM was utilized to determine the role of IL-8 in regulation of tumor metastasis and progression.

**Results:**

The elevated sIL-8 levels were associated with LNM and poor prognosis in gastric cancer. Furthermore, sIL-8 was identified to be prominently produced by gastric cancer-associated fibroblasts (CAFs). Elevated IL-8 can up-regulate PD-1 expression in CD8^+^ T cells, resulting in immunosuppression in primary tumors and TDLNs, which enhances LNM of gastric cancer. Molecularly, IL-8 increases PD-1 expression through JAK2/STAT3 signaling activation, and inhibits its ubiquitination via Fbxo38 down-regulation. In addition, the in vivo studies in mouse gastric cancer model demonstrated that IL-8 promotes LNM via PD-1 up-regulation in CD8^+^ T cells.

**Conclusion:**

The present study elucidates the pro-metastatic role of elevated IL-8 in gastric cancer, and provides novel insights to enhance immune checkpoint blockade therapy for anti-PD-1 in gastric cancer.

**Supplementary Information:**

The online version contains supplementary material available at 10.1007/s00262-022-03223-3.

## Background

Tumor-draining lymph nodes (TDLNs) are the major route of metastasis in gastric cancer, and lymph node metastasis (LNM) serves as one of the most important risk factors for poor prognosis [1]. However, the mechanisms underlying LNM remain uncertain [2, 3]. Tumor LNM is a series of concurrent, usually overlapping processes, involving lymphangiogenesis, pro-metastatic niche formation in TDLNs, tumor cells’ entry into TDLNs, escaping from immune attack and proliferation in TDLNs, and eventually formation of metastasis [4]. T lymphocytes act as the major effector cells for anti-tumor immunity, and exert immune surveillance and cytotoxicity through identifying tumor cells to inhibit tumor progression [5, 6]. A single cell analysis with naive lymph nodes and TDLNs in a mouse spontaneous breast cancer model revealed up-regulated angiogenesis pathway genes and high regulatory T (Treg)-associated genes, and down-regulation of interferon response and inflammatory response gene signatures in both CD4^+^ and CD8^+^ T cells within TDLNs, suggesting presence of an immunosuppressive microenvironment in TDLNs. This immune reprogramming of TDLNs during breast cancer progression is deemed to be key alterations in the preparation of pre-metastatic niche [7]. In addition, comparing with healthy lymph nodes, breast cancer-induced immune suppression occurs in the tumor-involved sentinel lymph nodes (SLNs), as evidenced by increased rates of Tregs or myeloid derived suppressor cells (MDSCs) as well as by generation of the dysfunctional state of T cells [8]. Therefore, we hypothesize that LNM of gastric cancer may also be associated with T cell dysfunctionality in TDLNs [9].

T cells may differentiate into different functional states following various antigen stimulations after entry into tumors or TDLNs [10]. It is well recognized that tumor cells may induce T cells to differentiate into inhibitory T cells such as Tregs by secreting IL-10 via exogenous regulatory pathways, and/or promote T cells to express inhibitory receptors such as programmed cell death-1 (PD-1) via endogenous pathways [11, 12]. T cell exhaustion is one of the most important mechanisms for immunosuppression [13, 14]. The hallmarks of T cell exhaustion include up-regulation of inhibitory receptors on T cells [15], reduced ability to produce effector molecules [16], and phenotypic changing of Tregs [13]. Interaction of the increased inhibitory receptors with the corresponding ligands on tumor cells or antigen-presenting cells (APCs) activates the negative immune regulation pathway, resulting in tumor immune evasion [11]. The prevalent inhibitory receptors on T cells comprise PD-1, lymphocyte activation gene 3 protein (LAG3), T cell immunoglobulin mucin receptor 3 (TIM3), and cytotoxic T lymphocyte-associate antigen 4 (CTLA4) [17, 18]. Among them, PD-1, by interacting with programmed death ligand 1 (PD-L1) or PD-L2, acts as a negative co-stimulatory receptor to induce T cell exhaustion. PD-1/PD-L1 axis is the main mechanism of tumor cells to escape anti-tumor immune reaction [19]. Immune checkpoint blockade (ICB) therapy targeting PD-1/PD-L1 has achieved definite and lasting efficacy in certain tumor types, including melanoma and lung cancer [20].

TDLNs also present T cell exhaustion [21]. Recent studies suggested that tumor specific antigen of melanoma could differentiate CD8^+^ T cells into PD-1^+^Tcf1^+^Tim3^−^ cells in TDLNs, resulting in immunosuppression with relative to non-draining lymph nodes (NDLNs) [22]. Furthermore, prevalence of PD-1 in CD8^+^ T cells in TDLNs was much higher than that in NDLNs, which was negatively related to survival in mouse tumor models. Anti-PD-1 treatment increased the total number of CD8^+^ T cells in TDLNs [23]. PD-1 or PD-L1 has also been shown to express in gastric cancer [24, 25]. Thus, we postulated that T cell dysfunctionality may be also presented in TDLNs of gastric cancer, which may play an important role in tumor LNM.

We have previously demonstrated that the pretherapeutic (or untreated) serum interleukin-8 (sIL-8) levels are elevated in about half of patients with locally advanced gastric cancer, and elevated sIL-8 levels are associated with Lauren classification, T staging and chemoresistance [26]. In the present study, we revealed that the elevated sIL-8 levels are related to LNM as well as poor tumor prognosis. Furthermore, sIL-8 was prominently produced by gastric cancer-associated fibroblasts (CAFs). To clarify the potential mechanisms underlying IL-8-mediated LNM, we performed profound bioinformatics analysis, and systematically assayed the CD4/CD8 ratio and PD-1 expression of T cells in primary tumor tissues, TDLNs and NDLNs, and evaluated their relevance with elevated IL-8 levels. It was showed that elevated IL-8 up-regulates PD-1 expression in CD8^+^ T cells, and subsequently results in immunosuppression in primary tumors and TDLNs, which enhances incidence of LNM. We also demonstrated that IL-8 increases PD-1 expression through JAK2/STAT3 signaling activation, and inhibits PD-1 ubiquitination via Fbxo38 down-regulation in CD8^+^ T cells. Furthermore, the mouse model study of gastric cancer with lymph node metastasis demonstrated that IL-8 promotes LNM via PD-1 up-regulation in CD8^+^ T cells. Our present study elucidates the pro-metastatic roles of elevated sIL-8 in gastric cancer, and provides novel insights to enhance immune checkpoint blockade therapy against PD-1 in gastric cancer.

## Materials and methods

Multiple materials and methods see the *Supplementary materials and methods*.

### Human serum and tissue specimens of gastric cancer patients

A consecutive series of 146 patients diagnosed with primary locally advanced gastric carcinoma from September 2019 to March 2020 at Department of Surgical Oncology, Affiliated Hospital of Nanjing University of Chinese Medicine, were enrolled in this study. All these patients were pathologically diagnosed as stomach adenocarcinoma following the American Joint Committee on Cancer (AJCC) criteria. All these patients had not undergone preoperative chemotherapy or radiotherapy. The serum specimens were collected preoperatively. The tumor tissues, the corresponding TDLNs and NDLNs were collected from all patients immediately after resection, and snap frozen in liquid nitrogen. The mesenteric lymph nodes of small intestine are set as NDLNs for stomach tumors according to the principle of lymphatic reflux. For flow cytometry assay, these specimens were transferred directly to the lab after resection. The samples were obtained following written consent according to the established protocol approved by the Institutional Review Board of Nanjing University of Chinese Medicine. This study was also in accordance with the Declaration of Helsinki.

### Cells

After the peripheral blood mononuclear cells (PBMCs) were isolated from healthy donors using Polymorphprep (1,114,683, Axis-Shield, Norway) with density gradient centrifugation, the CD4^+^ T cells and CD8^+^ T cells were further purified using anti-CD4 MicroBeads (130–045-101, MilteniyBiotec, Germany) and anti-CD8 MicroBeads (130–045-201, MilteniyBiotec, Germany) following the manufacturer’s instructions. All the cells were cultured in RPMI 1640 medium (Gibco, Grand Island, NY, USA) supplemented with 10% fetal bovine serum (FBS) (Gibco, Grand Island, NY, USA) and 1% penicillin/streptomycin (Gibco, Grand Island, NY, USA) at 37 °C. These isolated cells were stimulated with or without IL-8 (100 ng/ml, Peprotech, NJ, USA) and plate-bound anti-CD3/CD28 (5 μg/ml, Thermo Scientific, Waltham, MA, USA) in the presence of IL-2 (10 ng/ml) for the indicated time. All samples were collected with the informed consent of the donors, and all related procedures were approved by the Institutional Review Board of Nanjing University of Chinese Medicine. MFC, a mouse stomach adenocarcinoma cell line, was purchased from Shanghai Cafa Biological Technology Co. Ltd. (Shanghai, China).

### Establishment of lymph node metastasis model of gastric cancer in C57BL/6 mouse

Animal studies were approved by the Animal Care and Use Committee of Nanjing University of Chinese Medicine, and were in accordance with the principles and procedures outlined in the NIH Guide for the Care and Use of Laboratory Animals. C57BL/6 mice (4–6 weeks) were obtained from the Institute of Biomedical Sciences, Nanjing University. MFC cells were inoculated into mice (1 × 10^6^ cells *per* mouse) intraperitoneally. These mice were divided by random number method into three groups, and each contained 10 mice. On day 3 and day 7, exogenous IL-8 (AF-200-08 M, Peprotech, NJ, USA) was used intraperitoneally (10 μg *per* mouse) in the observation group and the treatment group, and isochoric PBS was used in the control group. On day 10, 13 and 17, anti-PD-1 mAb (aPD-1) (CD279) (BE0273, BioXCell, NH, USA) was administrated via caudal vein (200 μg *per* mouse) in the treatment group, and the IgG2a isotype (BE0089, BioXCell, NH, USA) was used in the observation group. On day 21, multiple tumors in abdominal cavity were collected after euthanasia, and the mediastinal and the inguinal lymph nodes were harvested simultaneously. According to the principle of lymphatic reflux, the mediastinal lymph nodes and the inguinal lymph nodes serve as TDLNs and NDLNs, respectively, for abdominal tumors in mice.

### Statistical analysis

GraphPad Prism software (version 8.0, La Jolla, CA) was used for statistical analysis. Independent Student *t*-tests were used to compare the continuous variables between two groups, and categorical variables were compared by the Chi-squared test. The Kaplan–Meier method and the Gehan-Breslow-Wilcoxon test were used to calculate the overall survival. All values in the text and figure were deviated by means ± standard error, and all experiments were repeated at least 3 times. *P* less than 0.05 was considered significant.

## Results

### Tumor-derived IL-8 is positively associated with lymph node metastasis and poor prognosis in locally advanced gastric cancer

**Fig. 1:**
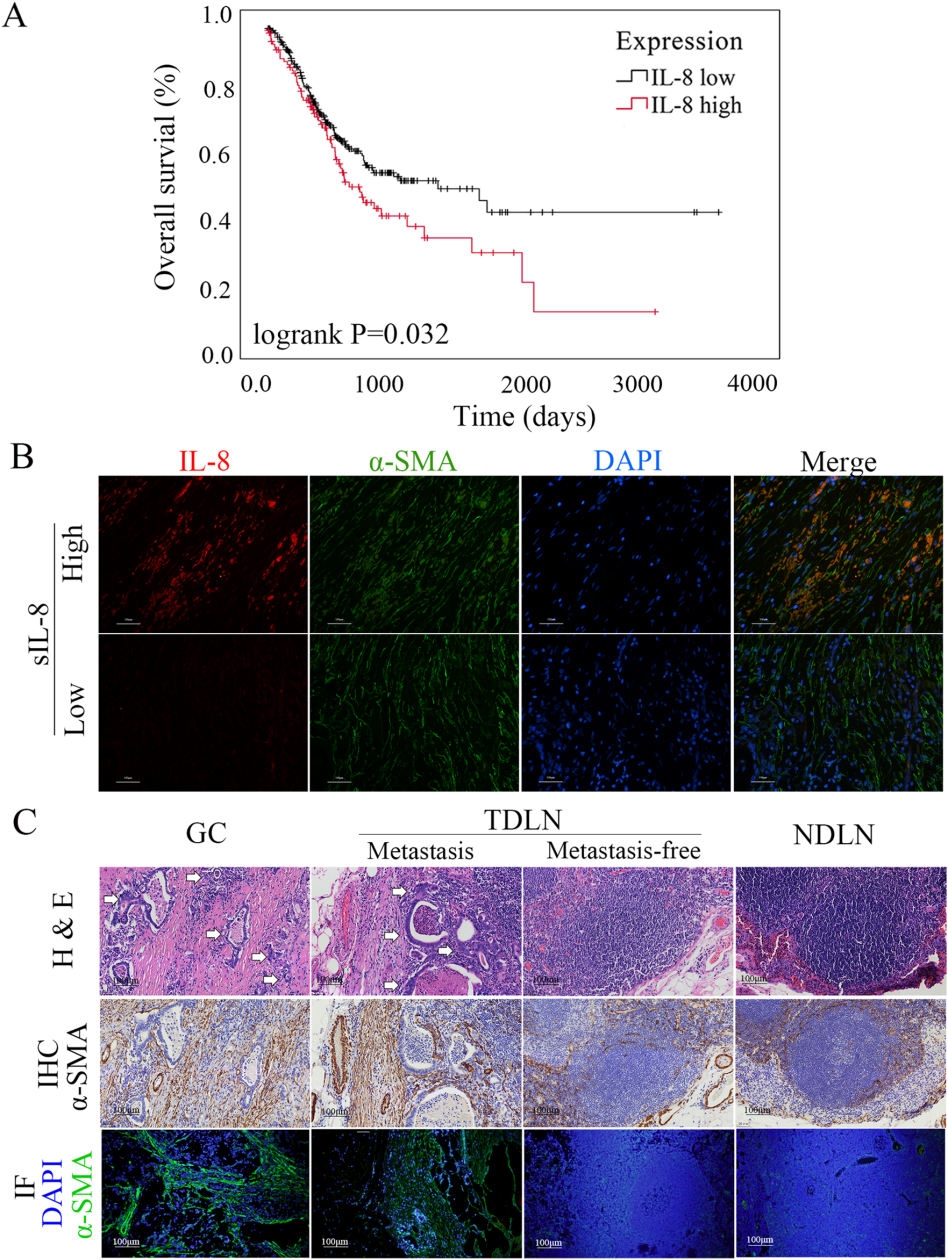
Tumor-derived IL-8 is associated with LNM and poor prognosis in locally advanced gastric cancer. **A** Study with the data from TCGA showed the higher IL-8 mRNA levels in tumor tissues were negatively associated with overall survival (OS) of gastric cancer patients (*P* = 0.032). **B** Immunofluorescence assay indicated IL-8 immunostaining was mainly detected in CAFs with α-SMA expression. **C** Lots of activated fibroblasts were detected using IHC and immunofluorescence (IF) assay in the TDLNs with tumor metastasis (white arrow), which is similar to that in the GC tissues (*P* = 0.450); however, few activated fibroblasts were found in the metastasis-free TDLNs and NDLNs

Previously, we revealed that approximately half of patients with locally advanced gastric cancer had elevated untreated sIL-8 level (> 62.00 pg/ml), and have investigated its clinicopathological relevance. We demonstrated that elevated sIL-8 is prominently produced by stomach tumors due to its dramatic decrease following radical gastrectomy [26]. To further identify the role of IL-8 in the pathogenesis of gastric cancer, we performed the present retrospective analyses. We uncovered that the elevated sIL-8 level served as the risk factor for lymph node metastasis (LNM) as well as Lauren classification and depth of tumor invasion (T staging), and that these three factors were also the independent factors for LNM in patients with locally advanced gastric cancer (Table 1). To address the potential prognostic significance of IL-8 in gastric cancer, the data from the Cancer Genome Atlas (TCGA), involving 421 gastric cancer patients, were analyzed and the cutoff of 10.425 was determined by receiver operating characteristic (ROC) curve analysis. We showed that the higher IL-8 mRNA levels in tumor tissues were negatively associated with overall survival (OS) of gastric cancer patients (*P* = 0.032, Fig. 1A).

**Table 1:** The risk factors for lymph node metastasis (LNM) in the enrolled gastric cancer patients

Factors	No	LNM (%)				*χ*^2^	*P*	Model fitting criteria	Likelihood ration tests		
		N0	N1	N2	N3			− 2 log likelihood	*χ*^2^	df	*P*
*Age (years)*						9.099	0.028	288.709	4.440	3	0.218
< 60	48	20 (41.7)	4 (8.3)	5 (10.4)	19 (39.6)						
≥ 60	98	33 (33.7)	14 (14.3)	28 (28.6)	23 (23.5)						
*Gender*						0.878	0.831	/	/	/	/
Male	101	38 (37.6)	12 (11.9)	24 (23.8)	27 (26.7)						
Female	45	15 (33.3)	6 (13.3)	9 (20.0)	15 (33.3)						
*Lauren classification*						27.076	0.000	303.535	19.266	6	0.004
Intestinal	54	29 (53.7)	8 (14.8)	12 (22.2)	5 (9.3)						
Diffuse	24	11 (45.8)	0 (0.0)	3 (12.5)	10 (41.7)						
Mixed	68	13 (19.1)	10 (14.7)	18 (26.5)	27 (39.7)						
*Depth of tumor invasion*						51.574	0.000	325.129	40.860	9	0.000
T1	36	26 (72.2)	5 (13.9)	2 (5.6)	3 (8.3)						
T2	19	10 (52.6)	2 (10.5)	5 (26.3)	2 (10.5)						
T3	67	16 (23.9)	10 (14.9)	20 (29.9)	21 (31.3)						
T4	24	1 (4.2)	1 (4.2)	6 (25.0)	16 (66.7)						
*Untreated sIL-*8 *level*						11.606	0.009	304.242	19.973	3	0.000
≤ 62 pg/ml	68	23 (33.8)	15 (22.1)	12 (17.6)	18 (26.5)						
> 62 pg/ml	78	30 (38.5)	3 (3.8)	21 (26.9)	24 (30.8)						

*sIL-*8 serum IL-8

Our previous IHC assays demonstrated IL-8 mainly located in cancer-associated fibroblasts (CAFs), and the primary CAFs produced more IL-8 than the respective primary normal fibroblasts (NFs) [26]. We further performed immunofluorescence (IF) assay and revealed that IL-8 immunostaining was exclusively detected in CAFs with α-smooth muscle actin (α-SMA) expression, which provided the direct evidence that sIL-8 was derived prominently from CAFs in gastric cancer tumor microenvironment (Fig. 1B). Collectively, these clinicopathological studies demonstrated that CAFs-derived IL-8 promotes LNM, and predicts dismal prognosis in gastric cancer. In addition, we also showed that lots of activated fibroblasts were detected in the TDLNs with tumor metastasis, which was similar to that in the GC tissues (*P* = 0.450); however, few activated fibroblasts were found in NDLNs and the metastasis-free TDLNs (Fig. 1C, Figure s1A).

### Bioinformatics analysis reveals presence of immunosuppression in tumor-draining lymph nodes as well as primary gastric cancers

**Fig. 2:**
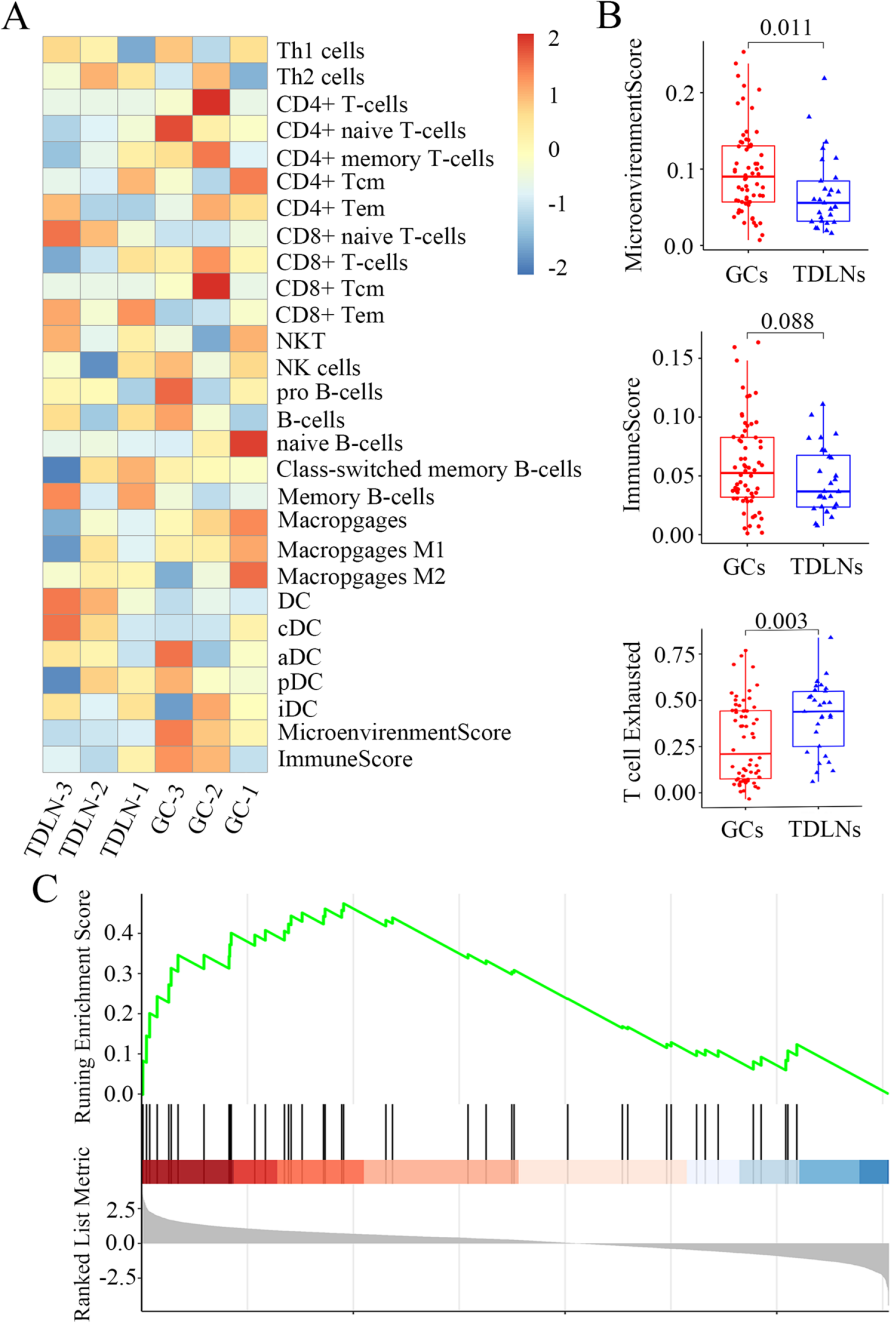
Bioinformatics analysis reveals presence of immunosuppression in tumor-draining lymph nodes as well as primary gastric cancers. **A** An overview of xCell scores for 28 immune cell types of GSE158631 in GEO database. CD8 + T cell enrichment in TDLNs was lower than in GCs, suggesting immunosuppression in TDLNs seems more remarkable than in tumor tissues. **B** The microenvironment score in TDLNs was lower than in GCs significantly (*P* = 0.011). The immune score in TDLNs were lower than in GCs, but it did not reach significance (*P* = 0.088). T cell exhaustion is more notable in TDLNs with relative to GCs (*P* = 0.003). **C** GSEA analysis identified PD-1 checkpoint pathway as one of the most significantly up-regulated pathways

Concerning the mechanisms underlying LNM in gastric cancer, we presumed it may be associated with immune status in primary tumors (GCs) and the respective TDLNs. We introduced xCell, a method to perform cell type enrichment analysis from gene expression data for 64 immune and stromal cell types (http://xCell.ucsf.edu/), to assess the immune microenvironment of GCs as well as TDLNs using the data in the GEO database (GSE158631) (https://www.ncbi.nlm.nih.gov/geo/query/acc.cgi?acc=GSE158631) [27], which analyzed comprehensively metastatic gastric cancer cells using single-cell RNA-seq. We showed CD8^+^ T cell enrichment in TDLNs was lower than that in GCs, suggesting that immunosuppression in TDLNs is more remarkable than that in tumor (Fig. 2A). Importantly, the microenvironment score (*P* = 0.011) and the immune score in TDLNs were lower than those in GCs, although the difference of immune score did not reach significant (*P* = 0.088). The average expression [measured by log2 (TPM + 1)] of 5 exhausted markers, including CTLA4, HAVCR2 (Hepatitis A virus cellular receptor 2), LAG3, PD-1, and TIGIT (T cell immunoreceptor with Ig and ITIM domains), was then used to define the exhausted score for CD8^+^ T cells. We found that T cell exhaustion is more notable in TDLNs with relative to GCs (*P* = 0.003) (Fig. 2B). Furthermore, gene set enrichment analysis (GSEA) performed by the Molecular Signature Database (MSigDB) was used to identify the potential regulators for immunosuppression in TDLNs and in primary tumors, and PD-1 checkpoint pathway was identified to be one of the most significantly up-regulated pathways (Fig. 2C), suggesting immunosuppression in primary gastric tumors and TDLNs is associated with PD-1 up-regulation.

### Elevated serum IL-8 levels are associated with CD8.^+^ T cell dysfunctionality in tumor-draining lymph nodes and promote LNM

**Fig. 3:**
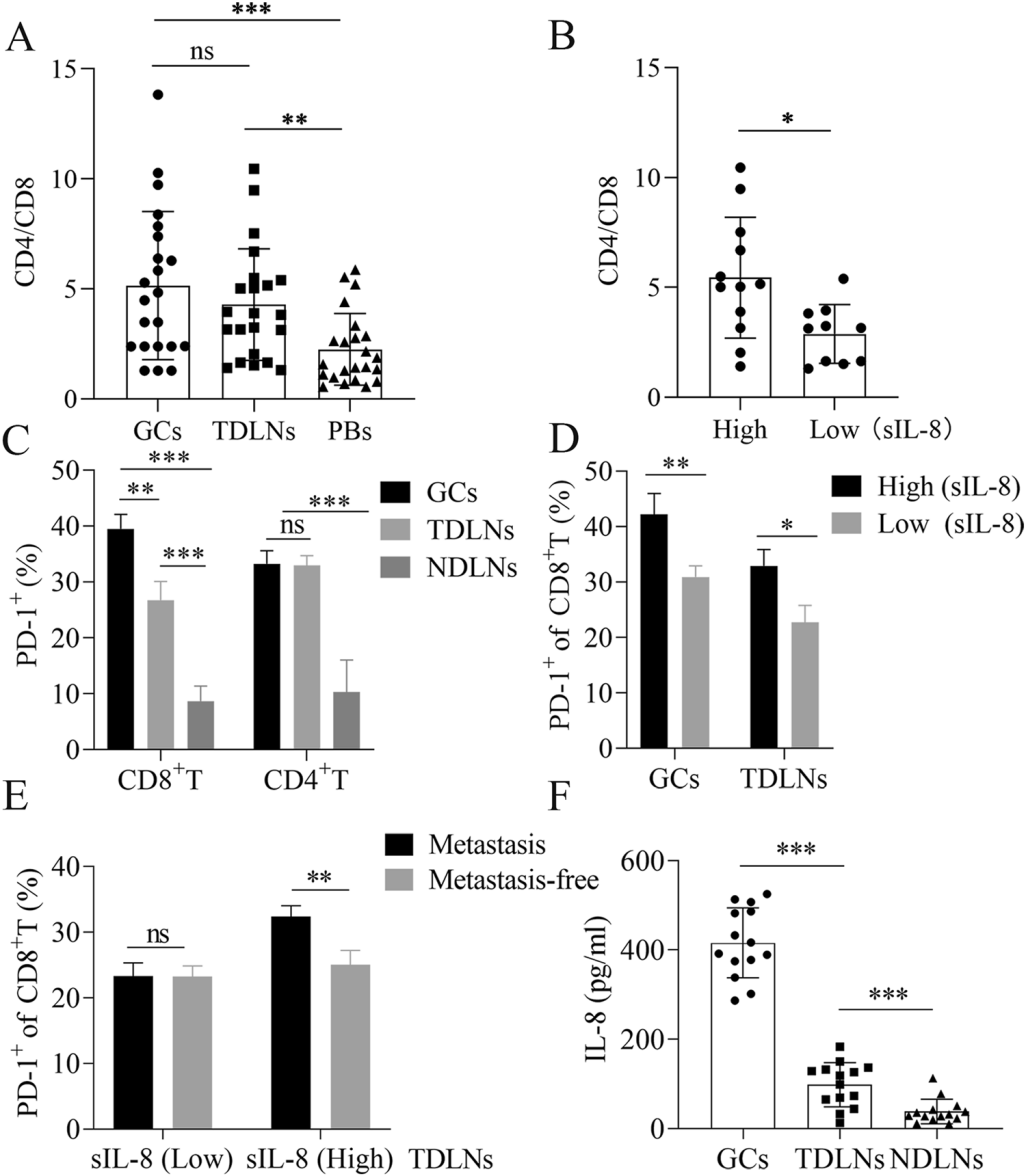
Elevated serum IL-8 levels are associated with CD8^+^ T cell dysfunctionality in TDLNS, and promote LNM. **A** The CD4/CD8 ratio of T cells in GCs or TDLNs was higher than in the PBs (*n* = 22). **B** In patients with elevated untreated sIL-8 level (*n* = 12), the CD4/CD8 ratio of T cells in TDLNs was higher than in patients with normal sIL-8 level (*n* = 10). **C** PD-1 prevalence in CD8^+^ T cells was higher in GCs than in TDLNs, but it had no difference in CD4^+^ T cells between GCs and TDLNs. PD-1 positive rate of CD4^+^ or CD8^+^ T cells was very low in NDLNs (*n* = 13). **D** In patients with elevated untreated sIL-8 level (*n* = 13), the percentage of PD-1^+^CD8^+^ T cells in both GCs and TDLNs were higher significantly than in patients with normal sIL-8 level (*n* = 11). **E** In patients with elevated untreated sIL-8 level (*n* = 15), the PD-1 positive rates of CD8.^+^ T cells in TDLNs with tumor metastasis were higher than in metastasis-free TDLNs, which was not found in patients with normal sIL-8 level (*n* = 13). **F** In 14 patients with elevated sIL-8 level, the IL-8 level was 455.76 ± 69.84 pg/ml, 92.41 ± 53.82 pg/ml and 23.41 ± 10.57 pg/ml in GCs, TDLNs and NDLNs, respectively. (* *P* < 0.05, ** *P* < 0.01, *** *P* < 0.001)

To verify the aforementioned bioinformatic analysis, we first analyzed the immune statuses in the primary tumor tissues (GCs), the corresponding TDLNs, and peripheral blood specimens (PBs) with FCM in patients with locally advanced gastric carcinoma. Intriguingly, the CD4/CD8 ratio of T cells in GCs or TDLNs was much higher than that in the PBs (*P* < 0.001 GCs *vs* PBs, *P* < 0.01 TDLNs *vs* PBs) (Fig. 3A), indicating the presence of local immunosuppression in GCs and TDLNs with relative to system immune status in gastric cancer patients. However, in patients with elevated untreated sIL-8 level (sIL-8 > 100 pg/ml), the CD4/CD8 ratio of T cells in TDLNs was much higher than that in patients with low sIL-8 level (sIL-8 < 62 pg/ml) (*P* = 0.0142) (Fig. 3B). These data suggested that the immunosuppression in TDLNs may attribute to elevated IL-8 levels.

**Fig. 4:**
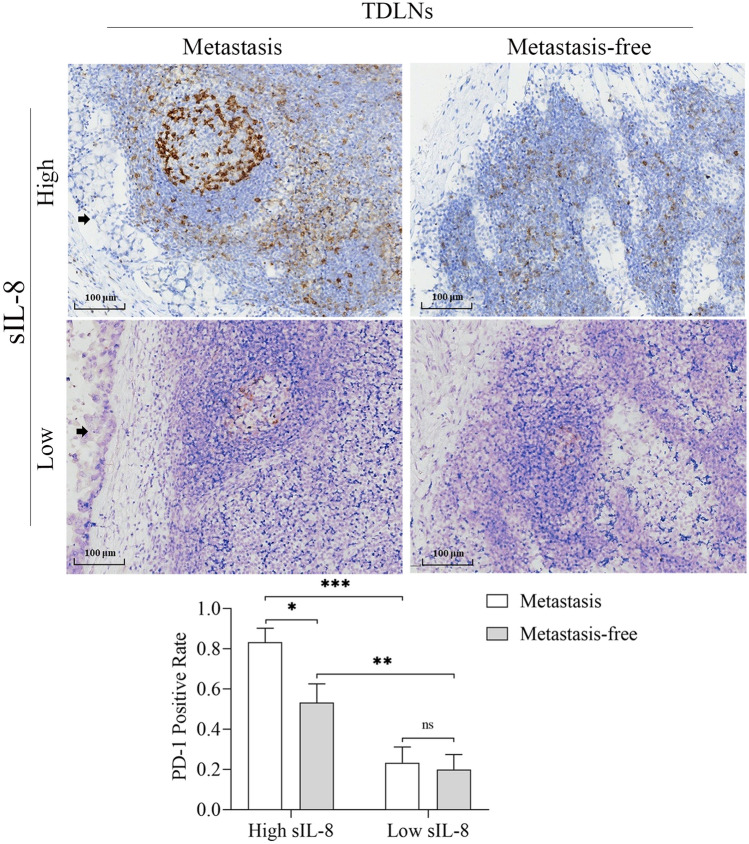
IHC assay for PD-1 in TDLNs of gastric cancer. IHC assay showed that more PD-1 immunoactivity was detected in patients with high sIL-8 level than in patients with normal sIL-8 level, and more PD-1 immunoactivity was found in TDLNs with tumor metastasis (black arrow) than in metastasis-free TDLNs. (* *P* < 0.05, ** *P* < 0.01, *** *P* < 0.001)

Next, we assayed PD-1 expression in T cells in GCs, the respective TDLNs and NDLNs in patients with locally advanced disease. We showed that PD-1 prevalence level in CD8^+^ T cells was much higher in GCs than that in TDLNs (*P* < 0.01), but it had no difference in CD4^+^ T cells between GCs and TDLNs. PD-1 positive rate of CD4^+^ or CD8^+^ T cells was very low in NDLNs (Fig. 3C). Importantly, in patients with elevated untreated sIL-8 level, the percentage of PD-1^+^CD8^+^ T cells in both GCs and TDLNs were higher significantly than that in patients with low sIL-8 level (*P* < 0.01 for GCs, *P* < 0.05 for TDLNs) (Fig. 3D). Furthermore, in patients with elevated untreated sIL-8 level, the PD-1 positive rates of CD8^+^ T cells in TDLNs with tumor metastasis were higher than that in metastasis-free TDLNs (*P* < 0.01), which was not found in patients with low sIL-8 level (Fig. 3E). IHC for PD-1 staining in TDLNs further approved these results (Fig. 4). These findings further supported the role of IL-8 in locally immunosuppressive status and in promotion of LNM.

To identify the correlation between IL-8 and the CD8^+^ T cell dysfunctionality in TDLNs and GCs, we assayed the IL-8 levels of GCs, TDLNs and NDLNs using the homogenate solutions of these tissues in 14 patients with elevated sIL-8 level, and showed that it was 455.76 ± 69.84 pg/ml, 92.41 ± 53.82 pg/ml and 23.41 ± 10.57 pg/ml in GCs, TDLNs and NDLNs, respectively (*P* < 0.001 GC *vs* TDLNs or NDLNs, *P* < 0.001 TDLNs *vs* NDLNs) (Fig. 3F).

Taken together, these data indicated that tumor-derived IL-8 is associated with PD-1 up-regulation in CD8^+^ T cells and immunosuppression in primary tumors and TDLNs, and promotes LNM in gastric cancer.

### IL-8 up-regulates PD-1 expression in CD8.^+^ T cell via STAT3 signaling activation, and inhibits PD-1 ubiquitination via Fbxo38 down-regulation

**Fig. 5:**
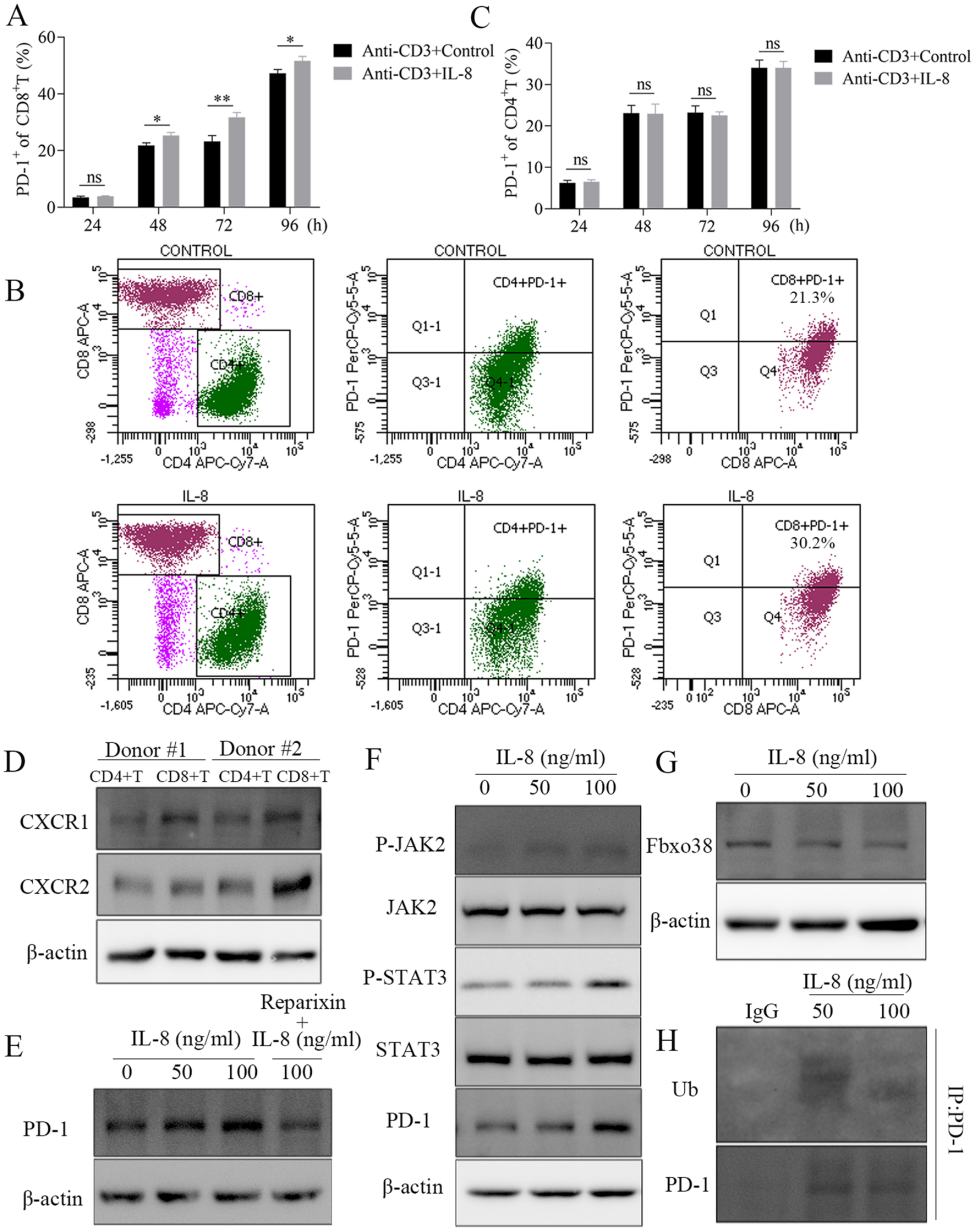
IL-8 up-regulates PD-1 expression in CD8^+^ T cell through JAK2/STAT3 signaling pathway, and inhibits PD-1 ubiquitination via Fbxo38. **A** IL-8 (100 ng/ml) treatment increased PD-1 positive rate in CD8^+^ T cells in time-dependent manner **B**, but it could not change PD-1 positive rate in CD4^+^ T cells **(C)**. **D** Higher level of CXCR1/2 was detected in CD8^+^ T cells than in CD4^+^ T cells. **E** IL-8 (96 h) treatment increased PD-1 level in CD8 + T cells in dose-dependent manner, which could be abrogated by reparixin. **F** IL-8 (96 h) treatment increased pJAK2 and pSTAT3 expression as well as PD-1 expression in dose-dependent manner. **G** IL-8 (96 h) treatment decreased Fbxo38 levels in CD8.^+^ T cells in dose-dependent manner. **H** CO-IP study indicated that PD-1 ubiquitination was inhibited by IL-8 treatment (96 h). (* *P* < 0.05, ** *P* < 0.01)

Next, we investigated the effects of IL-8 on PD-1 expression of T cells in vitro. We showed that IL-8 treatment (100 ng/ml) increased PD-1 positive rate in CD8^+^ T cells in a time-dependent manner (Fig. 5A and B), but it did not change PD-1 positive rate in CD4^+^ T cells (Fig. 5C). To address the underlying causative mechanism, the expression level of IL-8 receptor, CXCR1/2, was evaluated in primary CD4^+^ or CD8^+^ T cells. We found that higher level of CXCR1/2 was detected in CD8^+^ T cells than that in CD4^+^ T cells (Fig. 5D, Figure s1B), and that IL-8 treatment increased PD-1 level in CD8^+^ T cells, which could be abrogated by reparixin (8 μg/ml), a CXCR1/2 inhibitor (Fig. 5E, Figure s1C). More importantly, IL-8 treatment increased phosphorylated JAK2 (pJAK2) and pSTAT3 expression as well as PD-1 expression in a dose-dependent manner, indicating that IL-8 promotes PD-1 expression via activating JAK2/STAT3 signaling pathway (Fig. 5F, Figure s1D).

It has been shown that PD-1 may be degraded by ubiquitination, and that Fbxo38, an E3 ligase of PD-1, can mediate PD-1 ubiquitination [28]. We uncovered that IL-8 treatment could decrease Fbxo38 levels in CD8^+^ T cells in a dose-dependent manner (Fig. 5G, Figure s1E), and co-IP study indicated that PD-1 ubiquitination was inhibited simultaneously (Fig. 5H), which in turn resulted in PD-1 accumulation in CD8^+^ T cells.

Collectively, these results suggested that IL-8 up-regulates PD-1 expression in CD8^+^ T cell through activation of JAK2/STAT3 signaling, and inhibits PD-1 ubiquitination via Fbxo38 down-regulation.

### IL-8 promotes LNM of gastric cancer through up-regulation of PD-1 in CD8.^+^ T cells in vivo

**Fig. 6:**
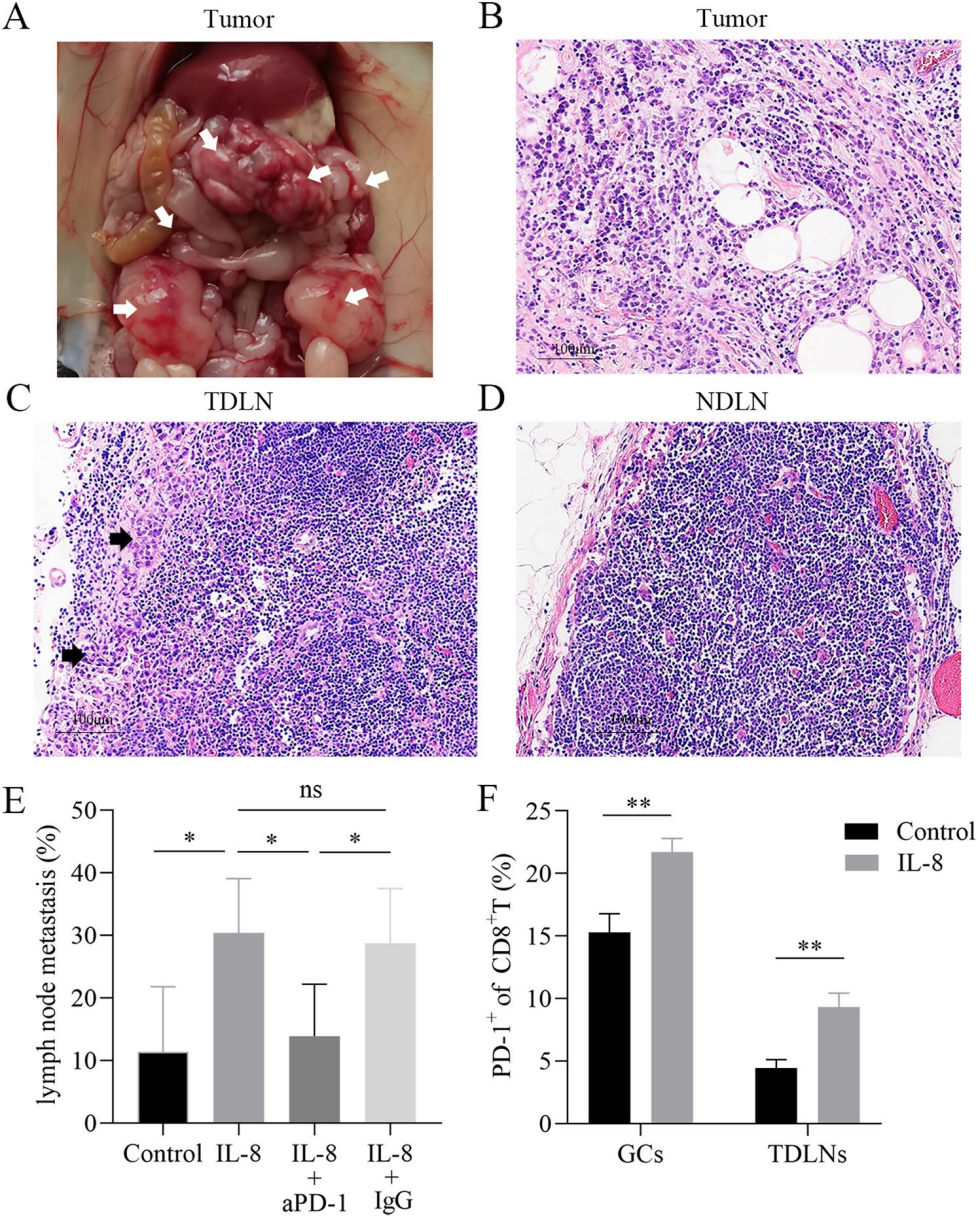
IL-8 promotes LNM of gastric cancer via up-regulation of PD-1 in CD8^+^ T cells in vivo. **(A)** MFC cells were inoculated into C57BL/6 mice (1 × 10^6^ cells *per* mouse) intraperitoneally, and abdominal tumors (white arrows) formatted, which was verified by pathological assay **(B)**. **C** The incidence of mediastinal LNM was increased in the observation group with relative to the control group, and aPD-1 administration reduced LNM frequency in the treatment group compared with the observation group. **D** IL-8 treatment enhanced PD-1 positive rate of CD8.^+^ T cells in abdominal tumors and in TDLNs. (* *P* < 0.05, ** *P* < 0.01)

Furthermore, we probed the role of exogenous IL-8 on LNM in vivo. MFC cells were inoculated into C57BL/6 mice intraperitoneally and established the mouse model of gastric cancer with lymph node metastasis. As shown in Fig. 6A and B, pathological assay (H & E) verified the abdominal tumor formation. About 3–5 mediastinal lymph nodes and 2–3 inguinal lymph nodes were harvested *per* mouse. Tumor metastasis could be detected in part of the mediastinal lymph nodes (Fig. 6C), but tumor cells could not be found in the inguinal lymph nodes (Fig. 6D).

Following exogenous IL-8 treatment, we showed that the incidence of mediastinal LNM was increased significantly in the observation group with relative to that in the control group (*P* < 0.05), and anti-PD-1 administration reduced LNM frequency in the treatment group compared with the observation group (*P* < 0.05) (Fig. 6E). Importantly, IL-8 treatment enhanced PD-1 positive rate of CD8^+^ T cells in abdominal tumors and in TDLNs (*P* < 0.01) (Fig. 6F), but IL-8 had no effects on PD-1 prevalence in CD4^+^ T cells (data not shown). The differences of abdominal tumors in these three groups were not observed, including number and volume.

Thus, it can be established that IL-8 promotes LNM of gastric cancer through up-regulation of PD-1 in CD8^+^ T cells in vivo, and anti-PD-1 therapy helps to inhibit LNM.

## Discussion

In the present study, we further demonstrated that elevated serum IL-8, mainly derived from cancer-associated fibroblasts, is associated with lymph node metastasis and poor prognosis in gastric cancer. Importantly, we unveiled the presence of locally immunosuppressive niche in primary tumors and TDLNs, which attributes to elevated IL-8 levels. This local suppression promotes LNM. IL-8 can increase PD-1 expression in CD8^+^ T cells but not in CD4^+^ T cells, and in turn promotes LNM in vivo, which can be obviated by blockage of PD-1.

LNM remains the main risk factor for prognosis in gastric cancer, but its mechanism is still unclear. Conceivably, LNM is associated with tumor microenvironment (TME). TME is a complex network formed by stromal, immune and inflammatory cells and the extracellular matrix (ECM). It has been well recognized that the development of metastases in TDLNs is associated with loss of immunogenicity or the tolerizing effects of the primary tumor cells, as well as with local immunosuppressive environment in TDLNs [9, 29]. Several mechanisms of tolerance have been revealed in experimental models and cancer patients as well, e.g. the effect of tolerogenic dendritic cells expressing indoleamine 2,3-dehydrogenase (IDO), or that of Tregs shown to accumulate in TDLNs [9].

The profound bioinformatic analysis of differentially expressed genes between gastric tumor tissues and TDLNs suggested the presence of immunosuppression in TDLNs as well as primary tumors, and we also showed TDLNs seem to possess more exhausted T cells than tumor tissues, which is associated with high PD-1 expression. PD-1 is an immune checkpoint molecule that is expressed on several types of immune cells, including effector T cells. It induces exhaustion of the effector function by engaging its ligand PD-L1 [30]. Several studies have reported the status of PD-1 or PD-L1 in gastric cancer, and PD-1 or PD-L1 has been shown to be potential predictive biomarker for recurrence and prognosis for gastric cancer in certain settings [31–33]. Our study approved the presence of locally suppressive microenvironment in tumor tissues as well as TDLNs, as evidenced by increased frequencies of PD-1^+^CD8^+^ T cells, which promotes LNM. Furthermore, IL-8 was shown to up-regulate the PD-1 expression in CD8^+^ T cells rather than CD4^+^ T cells, which may be due to the discrepancy of IL-8 receptors on CD4^+^ and CD8^+^ T cells. IL-8-mediated PD-1 up-regulation advanced LNM in vivo. However, we did not observe the significant alteration of PD-L1 expression in gastric cancer cell lines (data not shown). The role of the PD-1/PD-L1 pathway in promoting tumor immune evasion represents the rationale behind the development of monoclonal antibodies targeting either PD-1 or PD-L1 for cancer immunotherapy. Blocking the binding of PD-1 to its ligands has the potentials to expose tumor cells to the immune activity of effector T cells, and restores an effective anti-tumor immune response [24, 25]. In this study, we also showed that anti-PD-1 treatment could abrogate LNM mediated by IL-8.

However, only limited studies are available to investigate the PD-1 or PD-L1 in metastatic foci, especially TDLNs so far. Son et al. [34] compared the tumor immune microenvironments (TIMEs) of primary gastric cancer and paired metastatic gastric cancer. The metastatic gastric cancer involved liver, abdominal wall and ovary, but without lymph nodes. They showed TIME of metastatic tumors was less immunologically active compared to that of primary tumors in gastric cancer patients. Gao et al. reported that the positive rate of PD-1 on CD8^+^ T cells was significantly higher in primary tumors and metastatic lymph nodes than that in tumor-free lymph nodes, and that the intensity of PD-1 expression on CD8^+^ T cells in primary tumors and in metastatic lymph nodes were stronger than that in tumor-free lymph nodes from the same patient [35]. These findings are roughly consistent with our results.

Baseline serum IL-8 levels have been demonstrated to be associated with worse prognosis in advanced diseases across several tumor types. The elevated IL-8 levels are related to adverse tumor features, and can be used as a negative prognostic marker in patients treated with different therapies including immune-checkpoint inhibitors [36, 37]. IL-8 is a powerful chemoattractant for neutrophils and other potentially immune-suppressive myeloid leukocytes. Elevated IL-8 levels reflect a unique, unfavorable tumor microenvironment, which is characterized by prominent myeloid-cell infiltration, including neutrophils and monocytes, and limited adaptive T-cell responses. The unfavorable outcome is likely mediated by the negative immunomodulatory effects of elevated IL-8 levels [36]. Previously, we revealed that elevated untreated sIL-8 mediates chemoresistence via up-regulation of ABCB1 in gastric cancer [26]. We herein report a novel mechanism underlying IL-8-mediated immunosuppression in gastric cancer through inducing CD8^+^ T cell dysfunctionality, which expands the understanding of IL-8 roles in cancer.

In this study, the immunofluorescence assay provides further and direct evidence that IL-8 is mainly produced by CAFs in the gastric cancer tumor microenvironment in addition to our previous report [26]. However, mesenchymal stem cells (MSCs) or tumor-associated macrophages may also produce IL-8, and are involved in immune escape in gastric cancer [38, 39]. Due to the abundance of CAFs in TME and their ability to secret IL-8, we assume that sIL-8 is prominently derived from CAFs. We are conducting further studies to investigate the mechanisms underlying IL-8 up-regulation in CAFs. Interestingly, we found lots of activated fibroblasts in the TDLNs with tumor metastasis, rather than in the metastasis-free TDLNs and NDLNs, which may be involved in tumor lymph node dissemination. However, its molecular mechanisms remain to be investigated.

Certainly, it may be better to identify the subset of PD-1^+^CD8^+^ T cells involved in IL-8-mediated immune tolerance in gastric cancer, which could facilitate to potentially develop novel and precise strategies for treatment of gastric cancer metastasis. In addition, the detailed reinvigorating pathway of anti-PD-1 therapy for this situation also needs to be elucidated in the future.

## Conclusion

In summary, we report that tumor-derived IL-8 mediates PD-1 up-regulation in CD8^+^ T cells, resulting in a local immunosuppressive niche, which in turn facilitating LNM. Our study elucidates the novel pro-metastatic role of elevated IL-8 levels in gastric cancer, and provides new insights for immune checkpoint blockade therapy targeting PD-1 in gastric cancer.

## Supplementary Information

Below is the link to the electronic supplementary material.

Supplementary file1 (PDF 279 kb)

Supplementary file2 (PDF 820 kb) **Figure s1**. **A** Lots of activated fibroblasts were detected in the TDLNs with tumor metastasis, which is similar to that in the GC tissues (P = 0.450); however, few activated fibroblasts were found in the metastasis-free TDLNs and NDLNs. **B** Higher level of CXCR1/2 expression was detected in CD8+ T cells than in CD4+ T cells in these two healthy donors respectively. **C** IL-8 (96 h) treatment increased PD-1 level in CD8^+^ T cells in dose-dependent manner, which could be abrogated by reparixin. **D** IL-8 (96 h) treatment increased pJAK2 and pSTAT3 expression as well as PD-1 expression. **E** IL-8 (96 h) treatment decreased Fbxo38 levels in CD8^+^ T cells in dose-dependent manner. (* *P* < 0.05, ** *P*< 0.01, *** *P*< 0.001).

## Data Availability

The datasets used and/or analyzed during the current study are available from the corresponding author upon reasonable request.

## Abbreviations

AJCC: American joint committee on cancer
APC: Antigen-presenting cell
CAFs: Cancer-associated fibroblasts
co-IP: Co-immunoprecipitation
CTLA4: Cytotoxic T lymphocyte-associate antigen 4
ELISA: Enzyme-linked immunosorbent assay
ECM: Extracellular matrix
FCM: Flow cytometry
HBSS: Hanks balanced salt solution
IDO: Indoleamine 2,3-dehydrogenase
IL-8: Interleukin-8
ICB: Immune checkpoint blockade
IHC: Immunohistochemistry
LNM: Lymph node metastasis
LAG3: Lymphocyte activation gene 3 protein
MDSC: Myeloid derived suppressor cell
NDLN: Non-draining lymph node
NFs: Normal fibroblasts
OS: Overall survival
PD-1: Programmed cell death-1
PD-L1: Programmed death ligand 1
PBs: Peripheral blood specimens
PBMCs: Peripheral blood mononuclear cells
Treg: Regulatory T cell
ROC: Receiver operating characteristic
SLN: Sentinel lymph node
α-SMA: α-Smooth muscle actin
TIM3: T cell immunoglobulin mucin receptor 3
TCGA: The cancer genome atlas
TDLN: Tumor-draining lymph node
TME: Tumor microenvironment
TIMEs: Tumor immune microenvironments
