# Supplementary material for: Tumor-derived IL-8 facilitates lymph node metastasis of gastric cancer via PD-1 up-regulation in CD8+ T cells
Source: Cancer Immunol Immunother. 2022 May 28;71(12):3057–70. doi: 10.1007/s00262-022-03223-3 (PMC9588474; doi:10.1007/s00262-022-03223-3)
Supplement: Supplementary file 1 — Supplementary file1 (PDF 279 kb) [file 262_2022_3223_MOESM1_ESM.pdf]

Supplementary materials and methods

Enzyme-linked immunosorbent assay (ELISA)

Human IL-8 ELISA Kit (EH005-96, ExcellBio, China) was used to measure IL-8 levels in the patient serums and tissue supernatants. For tumor and lymph nodes, these tissues were weighted and homogenized in ice-cold RIPA buffer (RIPA, Beyotime, China) with protease inhibitor cocktail (Roche Applied Science, Penzberg, Germany) (100μl/10g). Then, the tissue homogenate solutions were centrifuged for 15 min (12, 000 ×g, 4 ℃). The supernatants were collected and used for the quantification of total protein and IL-8 levels. The supernatants were diluted in proportion before loading for ELISA assays.

Flow cytometry (FCM)

Prior to FCM assay, the tissue samples were processed into single-cell suspensions, and PBMCs were isolated by density gradient centrifugation. For tissues, the samples were washed with Hanks Balanced Salt Solution (HBSS) three times, then were cut into 1~2 mm pieces and digested in GEXSCOPE Tissue Dissociation Solution (Singleron Biotechnologies, China) in a 15 ml centrifuge tube with continuous agitation 15 min at 37 ℃. Following digestion, cells were separated by 40-micron sterile strainers (Corning) and centrifuged for 5 min (500 ×g, 4 ℃). Then, cells were washed with red blood cell lysis buffer (BD Biosciences, Franklin Lakes, NJ, USA), centrifuged and washed again with PBS. Remove the supernatants and re-suspend the cells in stain buffer (BD Biosciences, Franklin Lakes, NJ, USA) for FCM analysis with the BD FACSAria II flow cytometer (BD Biosciences, Franklin Lakes, NJ, USA). The cells were stained according to standard protocols with the following antibodies: FITC anti-human CD45 antibody (368508, BioLegend, CA, USA), PE anti-human CD3 antibody (317308, BioLegend, CA, USA), APC/Cyanine7 anti-human CD4 antibody (300518, BioLegend, CA, USA), APC anti-human CD8 Antibody (344722, BioLegend, CA, USA) and PerCP-Cy™5.5 mouse anti-Human CD279 (PD-1) antibody (561273, BD Biosciences, Franklin Lakes, NJ, USA).

Immunofluorescence assay

The section thickness of paraffin-embedded samples is 4 mm. In 0.01M citrate buffer, antigen was extracted in a pressure cooker within 20 min. Then, in PBS containing 10% bovine serum albumin, the sections were blocked at room temperature for 2 h. After blocking, samples were incubated with primary antibodies specific for mouse anti-α-SMA (1:100) (#48938S, Cell Signaling Technology, MA, USA), rabbit anti-IL-8 (1:250) (ab106350, Abcam, Cambridge, UK) overnight at 4°C. Fluorescent secondary antibody was carried out for 1 h at room temperature. Cell nuclei were counterstained with DAPI (Sigma-Aldrich, MO, USA). Images were acquired on a Zeiss LSM510 confocal microscope (Oberkochen, Germany).

Immunohistochemistry (IHC)

Immunohistochemistry assays were conducted according to the standard protocols. Mouse monoclonal anti-PD-1 antibody (Zsgb-Bio, Beijing, China) and rabbit anti-α-SMA (Cell Signaling Technology, MA, USA) were used. The staining intensity of tumor cells and the percentage of positive cells were scored by two pathologists. Staining intensity: 0 for no staining, 1 for weak staining, 2 for moderate staining and 3 for strong staining. The percentage of positive immune cells: 1 for ≤ 10% positive cells, 2 for 1% - 50% positive cells, 3 for 51% - 75% positive cells and 4 for > 75% positive cells. If the multiply of the two scores > 3, positive IHC expression was defined.

Western blotting assay

Protein expression levels of the indicated molecules were detected using western blotting assay. The antibodies used for the analyses were as following: anti-PD-1 antibody (86163S, Cell Signaling Technology, MA, USA), anti-FBXO38 antibody (ab87729, Cambridge, UK), anti-CXCR1 antibody (ab124344, Cambridge, UK), anti-CXCR2 antibody (ab65968, Cambridge, UK), anti-JAK2 antibody (3230S, Cell Signaling Technology, MA, USA), anti-p-JAK2 antibody (3771S, Cell Signaling

Technology, MA, USA), anti-STAT3 antibody (9139S, Cell Signaling Technology, MA, USA), anti-p-STAT3 antibody (9145S, Cell Signaling Technology, MA, USA) and anti-β-actin antibody (3700S, Cell Signaling Technology, MA, USA).

Immunoprecipitation

After the $CD8^+$ T cells were purified from peripheral blood and treated with IL-8 at certain concentrations, cells were collected and lysed in co-immunoprecipitation (co-IP) buffer (20 mM Tris, pH 7.5, 150 mM NaCl, 1% Triton X-100, and 1 mM EDTA) with protease inhibitors (Roche Applied Science, Mannheim, Germany) for 30 min on ice. Then the supernatant was taken following 10 min centrifugation (12, 000×g, 4 ℃), and the supernatant was incubated with primary anti-PD-1 antibody and GammaBind Plus Sepharose (#17088601, GE Healthcare, Logan, UT, USA), following rocking gently overnight at 4 ℃. On next day, the mixture was pelleted and washed with cold 1× co-IP buffer for six times before subjected to analysis by western blotting.
