# Supplementary figures and images for: Tumor-derived IL-8 facilitates lymph node metastasis of gastric cancer via PD-1 up-regulation in CD8+ T cells
Source: Cancer Immunol Immunother. 2022 May 28;71(12):3057–70. doi: 10.1007/s00262-022-03223-3 (PMC9588474; doi:10.1007/s00262-022-03223-3)

# Li's Supplementary Figure 1

**A**

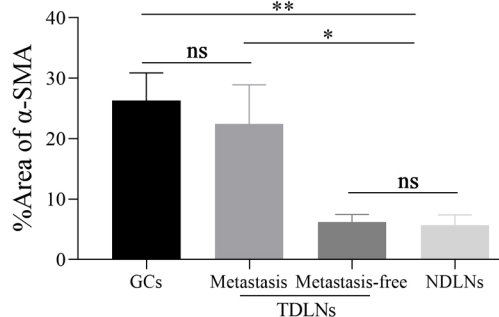

**C**

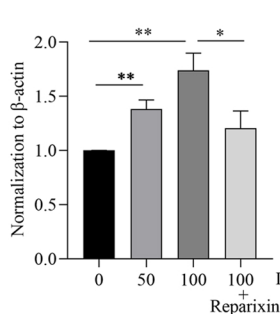

**E**

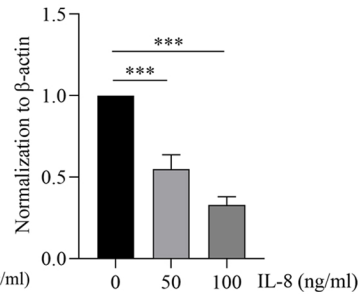

**B**

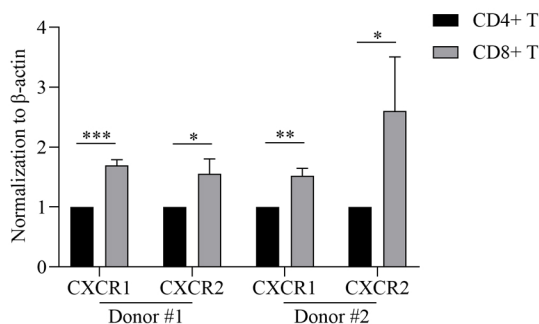

**D**

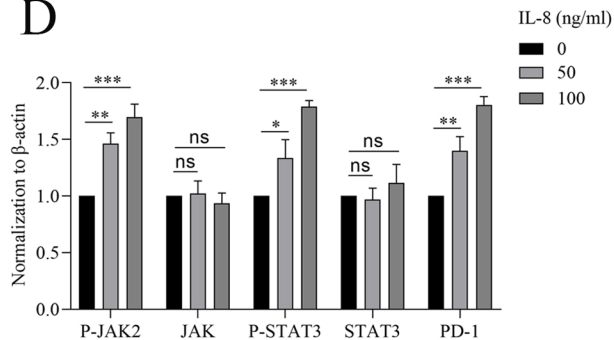

Supplement: Supplementary file 2 — Supplementary file2 (PDF 820 kb) Figure s1. A Lots of activated fibroblasts were detected in the TDLNs with tumor metastasis, which is similar to that in the GC tissues (P = 0.450); however, few activated fibroblasts were found in the metastasis-free TDLNs and NDLNs. B Higher level of CXCR1/2 expression was detected in CD8+ T cells than in CD4+ T cells in these two healthy donors respectively. C IL-8 (96 h) treatment increased PD-1 level in CD8+ T cells in dose-dependent manner, which could be abrogated by reparixin. D IL-8 (96 h) treatment increased pJAK2 and pSTAT3 expression as well as PD-1 expression. E IL-8 (96 h) treatment decreased Fbxo38 levels in CD8+ T cells in dose-dependent manner. (* P < 0.05, ** P< 0.01, *** P< 0.001). [file 262_2022_3223_MOESM2_ESM.pdf]
